# Metamorphosis and Gonad Maturation in the Horn Fly *Haematobia irritans*

**DOI:** 10.1673/031.011.17401

**Published:** 2011-12-31

**Authors:** Alicia L Basso, Natalia S. Forneris, Adrián Filiberti, Carlos E. Argaraña, Alejandro Rabossi, Luis A. Quesada-Allué

**Affiliations:** ^1^ Cátedra de Genética, Facultad de Agronomía, Universidad de Buenos Aires. Av. San Martin 4453, (1417) Buenos Aires, Argentina; ^2^ Centro de Investigaciones en Química Biológica de Córdoba (CIQUIBIC), Facultad de Ciencias Químicas-Universidad Nacional de Córdoba and Consejo Nacional de Investigaciones Científicas y Técnicas (CONICET), Ciudad Universitaria (5000), Córdoba, Argentina; ^3^ Instituto de Investigaciones Bioquímicas de Buenos Aires, CONICET, Fundación Instituto Leloir and Departamento de Química Biológica, Facultad de Ciencias Exactas y Naturales, Universidad de Buenos Aires. Patricias Argentinas 435, (1405) Buenos Aires, Argentina

**Keywords:** postembryonic development, Muscidae, cattle pest

## Abstract

The bloodsucking horn fly, *Haematobia irritans* (L.) (Diptera: Muscidae), is one of the most damaging pests of pasture cattle in many areas of the world. Both male and female imagoes spend their adult stage on the host, while immature stages develop in dung. Our goal was to determine if the progress of *H*. *irritans* gonad maturation can be correlated with eye and cuticle pigmentation events that occur during development of the imago within the puparium. The progression of germline cell divisions in immature gonads was analyzed from the beginning of the third larval instar (48 hours after egg hatch) until imago ecdysis. In the developing male larval gonad, meiosis began 72 hours after egg hatch, whereas in females oogonia were premeiotic at 72 hours. Meiosis was not detected in females until the mid-pharate adult stage, 120 hours after puparium formation. Therefore, gonad maturation in females appears to be delayed 144 hours with respect to that in males. In the stages within the puparium, the timing of germline cell division events was correlated with the progress of pigmentation of the eyes and cuticle as external markers.

## Introduction

*Haematobia irritans* (L.) (Diptera: Muscidae) is one of the most damaging pests of pasture cattle in many tropical and temperate areas of the world (Byford et al. 1992; Torres et al. 2002). Both male and female imagoes are haematophagous and spend their adult stage on the host. Females oviposit in freshly deposited cattle dung, where immature stages develop. *H*. *irritans* control has been primarily based on chemical insecticides; however, this has led to the development of resistance (Oyarzún et al. 2008). To find alternative methods of genetic sexing and control, new strategies must be developed that will require a better knowledge of the biology of this pest.

The sterile insect technique can only be used in certain restricted areas and requires massive production of insects, which is very difficult to implement with blood-sucking insects (Heinrich and Scott 2000). Alternative control methods of other dipterans, which have been developed and may be implemented, are growth regulators (Gillespie and Flanders 2009), autocidal control, lethal mutations, etc. (Bartlett and Staten 2009). Eventually, genetic strategies or substances specifically blocking *H*. *irritans* gonad development may be developed in the future.

Previous studies of male gonads of *H*. *irritans* imagoes have focused on chromosome number and morphology (LaChance 1964; Avancini and Weinzierl 1994; Parise-Maltempi and Avancini 2007). The apparent physiological age of *H*. *irritans* female imagoes has been determined by counting the number of nonfunctional ovarioles, among other characteristics (Schmidt 1972). However, to our knowledge, no information is available concerning early oogenesis in larvae, pupae, and pharate adult gonads of this insect. In particular, nothing is known about the onset of meiosis in either sex. Our goal was to determine if the progress of *H*. *irritans* gonad maturation could be correlated with eye and cuticle pigmentation events that occur during development of the imago within the puparium.

## Materials and Methods

### Collection of *H*. *irritans*

Adult *H*. *irritans* were collected with an entomological net from the backs of cattle and transferred by positive phototropism to *H*. *irritans* cages (15 × 15 × 25 cm), kept at 29 ± 1° C and fed with rags soaked with bovine blood with 0.05% sodium citrate to inhibit coagulation (Filiberti et al. 2009). The numbers of *H*. *irritans* per cage was approximately 1500.

### Larval rearing

Urine-free bovine faeces were employed as larval growth medium. Faeces were obtained immediately after deposition, from Aberdeen-Angus and Hereford cattle that were managed under natural grazing conditions. Since in laboratory conditions, optimal larval development required around 1g of bovine dung per egg (Lysyk 1991 and the researchers' previous data), individuals were seeded on the surface of 50g of dung.

Females were allowed to oviposit their eggs for 8 hours on pieces of cloth saturated with 8.5 g/l NaCl. The cloth was kept wet in a 90% humidity chamber for 12 h at 29° C until eggs hatched. Groups of 50 newly hatched 1^st^ instar larvae were seeded on the surface of faeces and kept in the dark, in a chamber at 29 ± 1° C. The age of each larvae was expressed in hours after egg hatch (h AEH) and larval stages were established under a binocular microscope.

**Figure 1.**
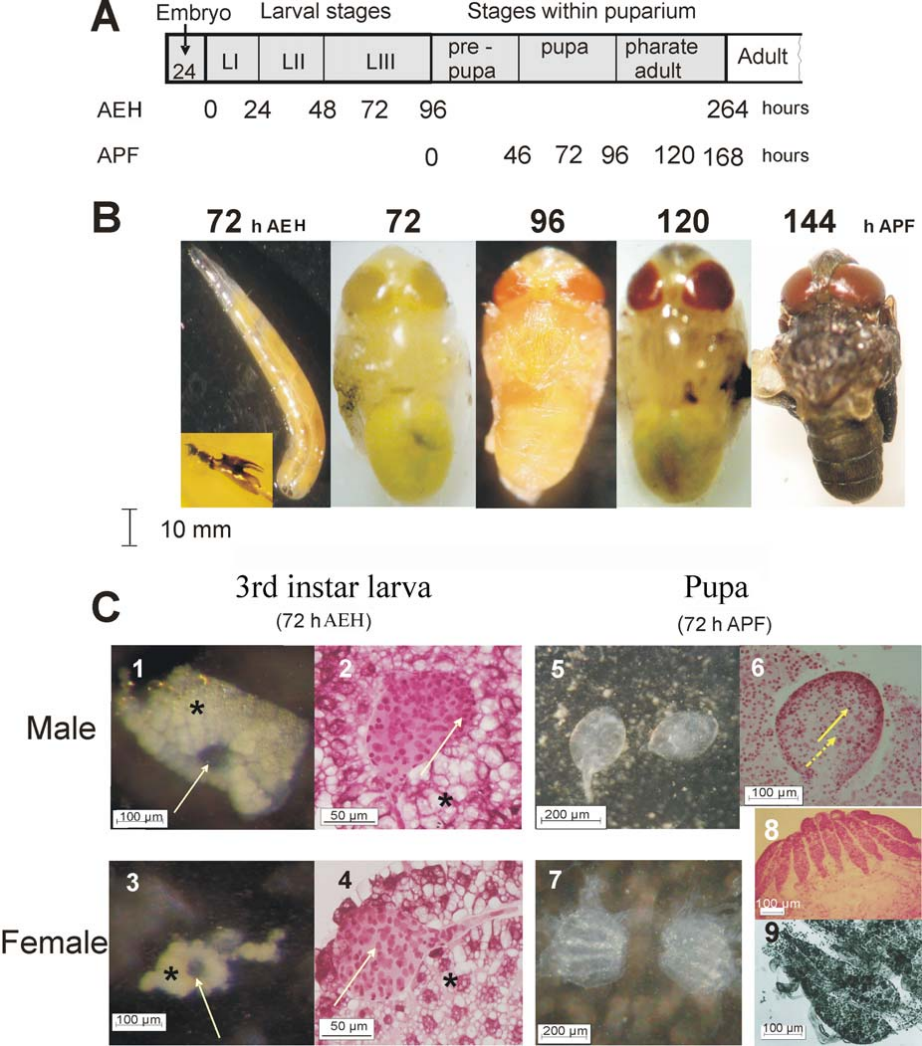
Postembryonic development of *Haematobia irritans* under laboratory conditions (29 ± 1° C and 90% RH). (A) Duration of larval stages and stages within puparium: age of the larvae is expressed in hours after egg hatching (h AEH). Age within the puparium is expressed in hours after definitive immobilization of the larva and onset of puparium formation (h APF). (B) Age-dependent phenotype: from left to right: 72 h AEH 3^rd^ instar larva with mouth-hooks amplified in the inset. Stages within the puparium showing the progress of eyes and cuticle pigmentation.: C (1–4): gonad development corresponding to 72 h AEH 3rd instar larva; C-1 and C-3 : arrows point to the gonad; * indicates fat body cells; C-2 and C-4: gonads staining (lacto-propionic orcein); C-2: arrow point to spermatogonia; C-4: arrow point to germline stem cells. C (5–9) Gonads stucture in 72 h APF pupae: C-5 testes and C-7 ovaries; C-6, C-8, and C-9: after staining and first squash. C-6: solid arrow point to spermatogonia; dashed arrow point to meiocytes I. C-8: different degree of ovarioles development within an ovary. C-9: growing cysts at the caudal region of the germarium. Amplifications used: C-1, C-3, C-5, and C-7: 40X. C-2 and C-4: 400X. C-6 and C-8:100X; C-9: 200X. High quality figures are available online.

The external morphology of the 3^rd^ larval stage has been described (Baker 1987). Independently of the size, the three larval stages were recognized by the shape the cephalopharyngeal skeleton (see the mouth hook in the inset of Figure 1B) and the posterior spiracles; as well as by the presence or absence of anterior spiracles (Ferrar 1979).

### Development within the puparium

Age within the puparium was expressed in hours after puparium formation (h APF), starting from the immobilization of the 3^rd^ instar (“untanned puparium”). Dissections cannot be made before 46–48 h without epidermis disruption. Therefore, the external features of the pre-pupal stage were not analyzed. As demonstrated for other dipterans, the criterion used to establish the onset of pupal and pharate adult stages was the deposition of the new cuticle, which can be assessed first by the synthesis and deposition of stage-specific cuticle proteins (Boccaccio and Quesada-Allué 1989) and, once the first layers of cuticle are deposited, by a very careful dissection (Rabossi et al 1991).

The pharate adult external morphology was recorded every 12 hours after separation of the puparium and pupal cuticle under a binocular microscope. The color of the eyes was determined using a Colour Atlas (Villalobos-Dominguez and Villalobos 1947). Lengths, times, and temperatures are expressed as means ± standard deviation.

### Gonad development.

**Figure 2.**
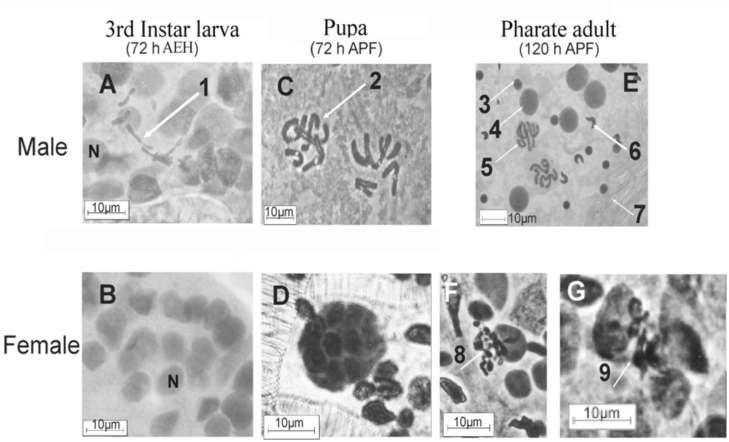
Gametogenesis in 3rd instar *Haematobia irritans* (72 h AEH), pupa (72 h APF) and pharate adult (120 h APF) stages. Images after second step-squash of 1C–2, 1C–4, 1C–6, and 1C–8. Male: A, C, E. Female: B, D, F, G. 3rd Instar larva- (A) Beginning of meiosis in spermatogonia (N= cell nuclei); arrow 1: spermatocyte I entering meiosis (prometaphase I ). (B) female stem cells in interphase (N = nuclei). Pupa- (C) spermatocyte in meiosis: arrow 2 points to a metaphase I. (D) female pre-meiotic cyst with 16 interconnected cells (further squash of preparation in Figure 1C–9). Pharate adult- (E) testis with nuclei in different stages of meiosis. Arrows indications: 3, meiocyte II; 4, meiocyte I; 5, metaphase II; 6, spermatids; and 7, spermatozoids. (F) Beginning of meiosis in oocyte I. Arrow 8 shows the karyosome stage. (G) oocyte in meiosis I, arrow 9 shows a metaphase I. (Bars indicate 10 µm; amplification 1000X). High quality figures are available online

Insects (N = 223) were dissected in Ringer's insect solution (Ashburner 1989). The nomenclature used by Ogienko et al. (2007) was employed when referring to development of the larval and pupal gonads. Sex determination in larvae was based on the spatial relationship between the gonads and the fat body as described by Demerec (1994). Developing male and female gonads from the 3^rd^ instar to the imago were dissected under a binocular microscope, and after digital recording (Sony Cyber-shot DSC-W100, www.sony.com) they were stained with lactopropionic orcein (color panels in Figure 1 and Figure 2) as described by Franceskin (2005). Cytological preparations (N = 135 out of 223 dissections) were obtained by a two-step progressive squashing of the tissue (Basso and Lifschitz 1995). First, light pressure was applied to squash and record the overall gonad structure. Then a second squash was applied to observe germline cells divisions, mitosis, and meiosis that were analyzed under an optical microscope (Zeiss, Axioplan, www.zeiss.com).

## Results

### Life cycle

In order to correlate the *H*. *irritans* postembryonic development with gametogenesis, a standard life cycle on cattle dung was established under laboratory conditions at 29 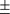 1° C and 90% relative humidity. Embryogenesis lasted 24 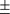 1 hours, whereas the full cycle until imago ecdysis lasted 12 days (Figure 1A). The span of larval development was 96 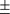 4 h AEH (Figure 1A). The mean length of the newly eclosed 1^st^ instar larvae was 1.5 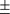 0.2 mm, attaining 6.0 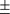 1.3 mm at the end of the 3^rd^ instar (92–94 h AEH). Then, the 3^rd^ instar migrated from the wet core of the dung to the drier border edge and began to retract the first three anterior segments to initiate pupariation; thus the 3^rd^ instar attained a final ovoid shape with a length of 4.5 ± 0.2 mm by 96 ± 4 h AEH = 0 h APF. Under the conditions of the present study, the stages within the puparium lasted 7 days, ending 168 ± 6 h APF. The span of the prepupal stage was 46 ± 2 h, the pupal stage lasted 50 ± 2h, and the span of the pharate adult stage was 72 ± 4h (Figure 1A).

### **Body markers and cuticle pigmentation**

Third instar larvae were recognized by posterior spiracles (Figure 1B) and mouth hook morphology (inset to Figure 1B). The pupal stage elapsed from the deposition of the new pupal cuticle at 46 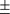
2 h APF until the deposition of the pharate adult cuticle, 96 ± 2 h APF. The evagination of the imaginal discs of head and thoracic appendages occurred at 48 ± 2 h APF. As expected, no pigmentation of cuticle structures in the new appendages was observed during the early pupal stage (not shown). Table 1 shows the timing of eyes and body markers pigmentation in late pupae and pharate adults.

**Table 1.**
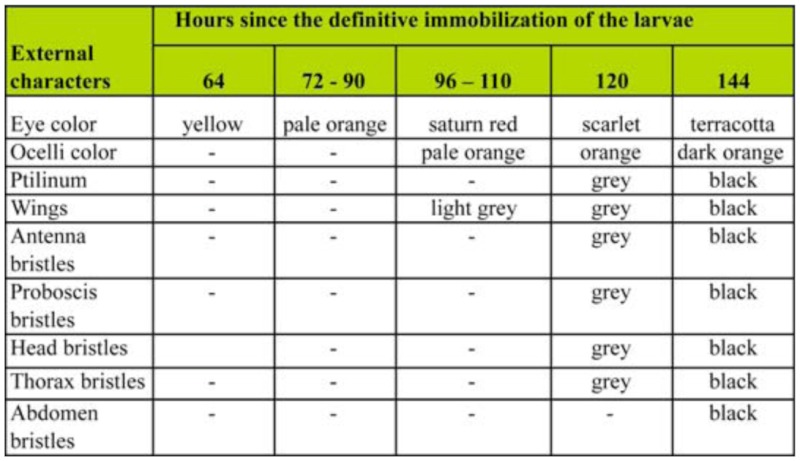
Pigmentation of external body structures of *Haematobia irritans* pupae and pharate adults.

During the pupal stage the colour of the eyes changed from pale yellow (Code: Y-19-12°) at 48 h APF to yellow (Y-18-12°) at 64 h APF, and attained a pale orange colour (Code: 0-17-8°) at 72 h APF, which then became progressively more intensely colored until the end of the stage (Figure 1B).

The transition from pupa to pharate adult occurred at 96 ± 2 h APF when the new cuticle was deposited. At this time, the eyes acquired a saturn red color (Code: SO-14-12°), whereas the ocelli pigmentation became evident (Figure 1B and Table 1). The thoracic hair became visible, but the cuticle showed very little or no pigmentation. After 96 h APF the wings were the first to show the onset of dark melanic pigmentation (light grey) (Table 1). Most of the head and thorax bristles, together with the ptilinum, initiated melanization between 110 and 120 h APF, when the colour of the eyes turned to scarlet (Code; SSO-10-12°) (Figure 1B). The eyes attained their definitive colour, terracotta (SSO-10-7°), and the ocelli became dark orange at 144 h APF, more than 20 h before ecdysis; whereas the color of the body acquired the typical very dark pigmentation (Figure 1B). The timing of eye pigment deposition and cuticle markers coloration was similar in both sexes.

### Developing larval gonads

Gonads in the early larvae were difficult to study. Developing male and female gonads from the beginning of early (72 h AEH) 3^rd^ instar were dissected (N=55). The location was four segments from the caudal end, i.e. at the level of segment A5. The size and the shape of the surrounding fat body were characteristic for each sex. Gonad cells were translucent, whereas fat body was more opaque (Figure 1C–1 and 1C–3). Male gonads of 72 h AEH larvae are ovoid (Figure 1C–1), carrying spermatogonial cells (Figure 1C–2). They were loosely attached to the surrounding fat body. Figure 1C–3 shows that female larval gonads were spherical, much smaller than male gonads, and lay tightly attached to, and embedded within, fat body cells in a rosette pattern carrying germline stem cells and cystoblasts (Figure 1C–4).

### Timing of gonads development in pupae and pharate adults

During the pre-pupal stage (0–46 h APF), tissue histolysis made the isolation of gonads difficult. A correlation between the timing of eye and external body markers pigmentation, described above, and gonad development was established during pupal and pharate adult stages. Results were highly reproducible in all the laboratory colonies initiated from repetitive field sampling carried out during the present experiments, and were also preliminarily confirmed in immature *H*. *irritans* collected in the field (not shown). From the beginning of the pupal stage (46 ± 2 h APF) until the establishment of pale orange eyes at 72–85 h APF, the observed pupal testes were ovoid and brilliant (Figure 1C–5) and showed gonial cells as well as populations of meiocytes I (Figure 1C–6). At 72 h APF, the well-formed ovaries of female pupae looked like a white shell (Figure 1C–7) and each ovary consisted of 9–12 ovarioles (Figure 1C– 8). Only a germarium seemed to be present and no follicles were visible (Figure 1C–8). Ovaries within a pair matured asynchronously, and within an ovary, ovarioles did not show the same degree of development. Some ovarioles showed premeiotic growing cysts at the caudal region of the germarium (Figure 1C–9). The cyst is a group of 16 interconnected cells derived from four mitotic divisions of the cystoblast.

### Onset of meiosis during gametogenesis

In male gonads of the mid-3^rd^ instar (72 h AEH) (Figure 1A), secondary spermatogonial cells in premeiotic interphase and primary spermatocytes in different stages of meiosis I up to metaphase I (N = 27) were detected. Figure 2A shows secondary spermatogonial cells and a pro-metaphase I in 3^rd^ instar larval testes (arrow 1 in Figure 2A). In the chronologically equivalent female larval gonad only pre-meiotic cystoblasts in the interphase stage were found (Figure 2B) (N = 28).

Figure 2C shows a metaphase I in a 72 h APF male pupal gonad. At 72 h APF, after resquashing the cytological preparation showed in figure 1C–8, only a pre-meiotic cyst formed by 16 interconnected cells or cystocytes was found in the ovariole of female pupal gonad (Figure 2D), marking the onset of the meiotic cell cycle; however, the onset of the first female meiosis was not detected until 115– 120 h APF. In addition, primary oocytes were observed, although most of the ovary maturation took place after the emergence of the imago. Some females showed the karyosome stage (Figure 2F); and images compatible with pro-metaphase I and metaphase I were observed, the latter being the phase of meiotic arrest (Figure 2G). In contrast, during the mid-pharate adult instar, male testes exhibited all the stages of spermatogenesis, including spermatids and sperm (Figure 2E).

During the first three days after female eclosion mature eggs were not present, in accordance with Schmidt (1972).

## Discussion

The success of every species depends on an efficient process of gametogenesis. Knowledge of the pattern of *H*. *irritans* gametogenesis is not merely of academic interest, but is also required to detect abnormal phenotypes that could eventually be used in strategies of genetic control (Heinrich and Scott 2000) in certain regions. Here a reproducible partial life cycle of *H*. *irritans* was established under laboratory conditions. This allowed the use of cuticle and eye color as useful developmental markers to be correlated with gametogenesis events, during stages within the puparium. This correlation was clearly established in the laboratory in several colonies generated from different wild populations. Thus, the onset of meiosis in male and female gonads was timed with sufficient accuracy.

The beginning of *H*. *irritans* male gametogenesis occurs during the 3^rd^ instar as observed among several cyclorrhaphan species (Table 2), with the apparent exception of *Oestrus ovis* in which spermatogenesis seems to be carried out mainly at the beginning of pupariation (Cepeda-Palacios 2001). However, the beginning of female gametogenesis was found to be variable among cyclorrhaphan flies, since a tendency to delay female meiosis seems to occur (Table 2). The onset of meiosis is a key point in the female gonad development. In *Anastrepha fraterculus* (Franceskin 2005) and *Hypoderma* spp (Boulard 1967; Scholl and Weintraub 1988), oogenesis occurs during the third instar, similarly to that observed in male spermatogenesis (Table 2). In the best—studied fly, *Drosophila melanogaster*, the delay between male and female meiosis has been documented by Demerec (1994) and Bolivar et al. (2006) (Table 2). This difference in maturation resembles our results for *C. capitata*, and for *Oestrus ovis*, where the female gametogenesis was estimated to occur during the transition from the pupa to pharate adult (Table 2) (Cepeda-Palacios et al. 2001).

**Table 2.**
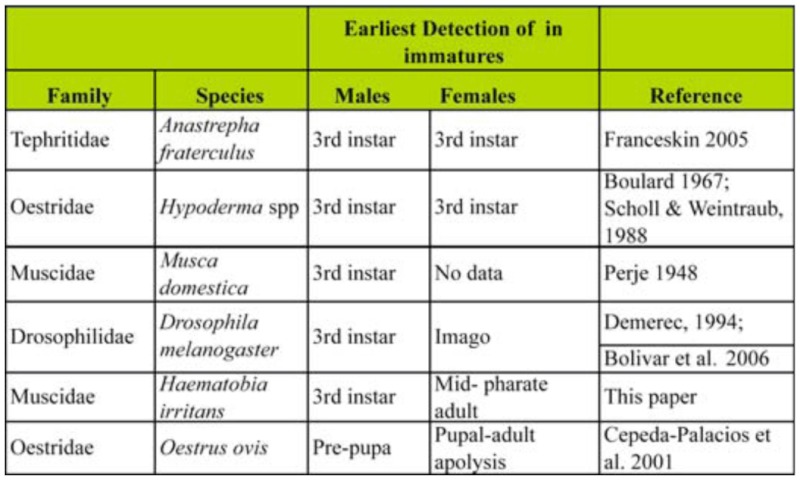
Onset of gametogenesis in *Haematobia irritans.* Comparative starting point recognition in different dipterans.

The present work shows clear evidence that the program of maturation of the ovary in *H*. *irritans* appears to be significantly delayed with respect to testis development. The difference observed in the appearance of meiotic structures between both sexes was 144 h, ranging from 72 h AEH in the 3^rd^ instar male to around 120 h APF in female pharate adults (Figure 1, Table 2). Thus, a sex— dependent, probably hormone—dependent, and differently timed endogenous clock seems to exist in germ cells. In general, a thorough understanding of all phases of gametogenesis is necessary before the effects of different levels of insectistatics (suppressants of growth or reproduction) or sterilizing agents can be assessed (WHO 1968). In the special case of *H*. *irritans*, further knowledge of the types of cells present in the testes and ovaries during development will be necessary before prospective insect sterilization studies can be properly conducted.

## **Abbreviations**

h AEH: hours after egg hatch
h APF: hours after puparium formation
